# Atypical Intracerebral Hemorrhage With a Fatal Outcome Following the ChAdOx1 nCoV-19 Vaccine

**DOI:** 10.7759/cureus.114440

**Published:** 2026-08-12

**Authors:** Mohamed Alem, Imane Kamaoui, Zayneb Alami

**Affiliations:** 1 Department of Pharmacy, Faculty of Medicine and Pharmacy, Mohammed VI University Hospital, Mohammed 1st University, Oujda, MAR; 2 Department of Radiology, Faculty of Medicine and Pharmacy, Mohammed VI University Hospital, Mohammed 1st University, Oujda, MAR

**Keywords:** case report, chadox1 ncov-19 vaccine, covid-19 vaccine, intracerebral hemorrhage, vaccine-induced adverse event

## Abstract

Intracerebral hemorrhage (ICH) is a severe form of stroke and is usually associated with established vascular risk factors. Neurological adverse events following ChAdOx1 nCoV-19 vaccination are uncommon, and isolated ICH occurring without thrombocytopenia or coagulation abnormalities is rarely observed. We report here the case of a 41-year-old woman, without cardiovascular risk factors or coagulation abnormalities and with no remarkable past medical history, admitted to our hospital with an afebrile altered level of consciousness that appeared 15 days after receiving her dose of ChAdOx1 nCoV-19 vaccine. She died two days after hospitalization (18 days after receiving the COVID-19 vaccine) due to ICH. This case is characterized by several atypical features, including fatal ICH occurring within a compatible timeframe following vaccination, in the absence of thrombocytopenia, coagulation abnormalities, or evidence of vaccine-induced immune thrombotic thrombocytopenia. It highlights the heterogeneity of neurological events occurring after vaccination and the importance of continued pharmacovigilance.

## Introduction

Intracerebral hemorrhage (ICH) accounts for 10-20% of all strokes, with a global incidence of approximately 20-25 per 100,000 person-years increasing with age. The risk is higher in men and Asian populations. Well-known risk factors include hypertension, cerebral amyloid angiopathy, anticoagulant use, and lifestyle factors such as alcohol and smoking. Despite declining trends in high-income countries, incidence remains high in low- and middle-income regions [1].

Rare but serious neurological complications have been reported following COVID-19 vaccination, particularly with viral vector vaccines such as ChAdOx1 nCoV-19 (Oxford-AstraZeneca). Among these, ICH has been reported, including in the context of vaccine-induced immune thrombotic thrombocytopenia (VITT), a rare condition characterized by thrombocytopenia and thrombosis following vaccination. However, isolated cases of hemorrhagic stroke have also been described in the absence of thrombocytopenia or other laboratory abnormalities suggestive of VITT. In particular, de Mélo Silva et al. reported a large intracerebral hemorrhage occurring five days after ChAdOx1 nCoV-19 vaccination, with normal platelet count, fibrinogen, prothrombin time, and D-dimer levels and no evidence of intracranial thrombosis or aneurysm. These events typically occur 5-12 days post-vaccination in adults without clear predisposing factors, and case reports suggest a higher incidence following ChAdOx1 nCoV‑19 than with other COVID‑19 vaccines [2,3].

Therefore, reporting similar cases is crucial to improve our understanding of these events, better identify potential associations and risk factors, and generate hypotheses for future research. In this context, we report a case of ICH following vaccination with the ChAdOx1 nCoV-19 vaccine in a 41-year-old Moroccan woman in the absence of VITT.

## Case presentation

A 41-year-old woman, a mother of three children, with no cardiovascular risk factors and an unremarkable medical history, was admitted to our hospital with an afebrile altered level of consciousness occurring 15 days after receiving her dose of ChAdOx1 nCov-19 vaccine (the dose number was not specified). The Glasgow Coma Scale (GCS) score was 3/15 on admission. Her blood pressure was 130/80 mmHg, with no history of anticoagulant use or traumatic event. No other relevant abnormality or evident alternative cause of the ICH was identified.

**Figure 1:**
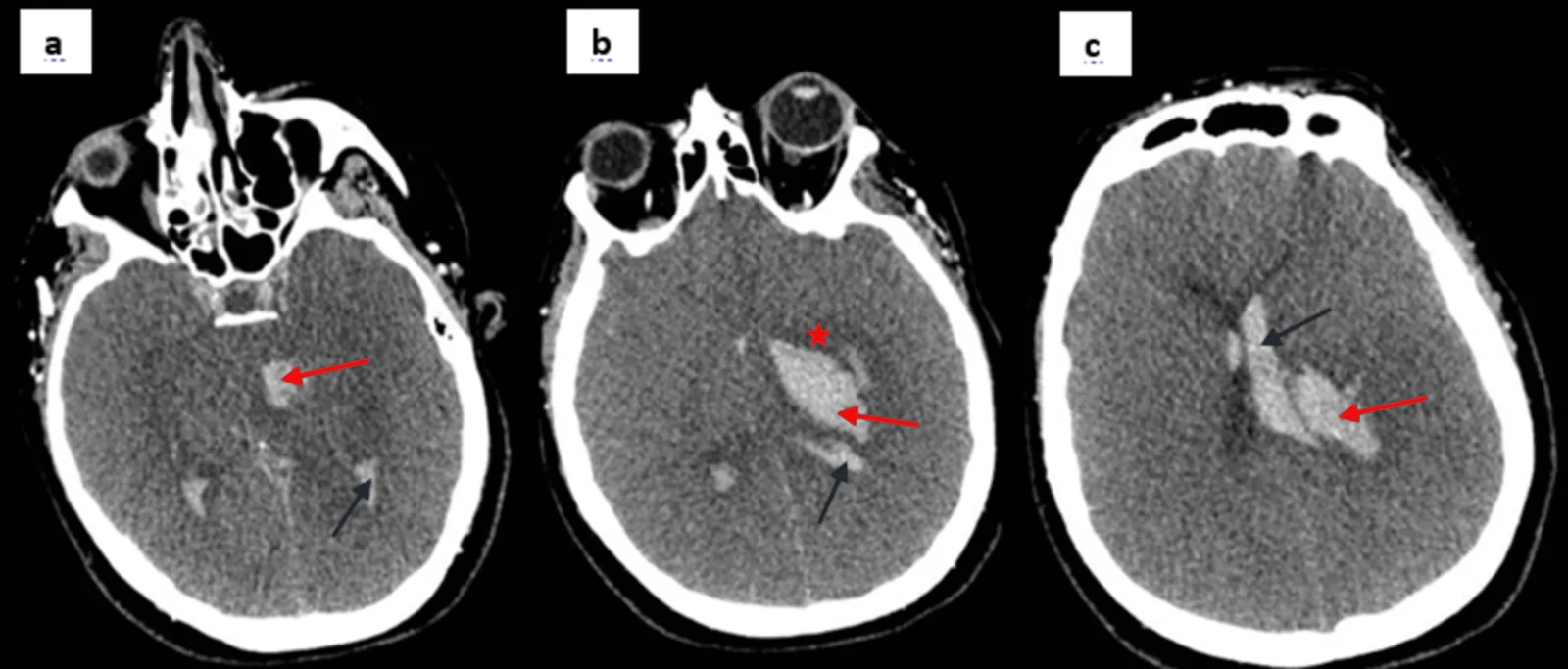
CT scan demonstrating a deep capsulolenticular intraparenchymal hemorrhage (red arrows in a, b, and c) extending into the brainstem, associated with extensive intraventricular hemorrhage (black arrows in a, b, and c), diffuse cerebral edema (red asterisk * in b), and subfalcine herniation.

Eight days after receiving her vaccine dose (one week before admission), the patient developed a headache associated with ocular pain and photophobia, which was unresponsive to paracetamol, followed by a progressive deterioration in consciousness. A brain computed tomography (CT) scan revealed a deep capsulolenticular intraparenchymal hemorrhage extending into the brainstem, associated with extensive intraventricular hemorrhage, diffuse cerebral edema, and subfalcine herniation, as shown in Figure 1.

The main hematological and biochemical laboratory findings of the patient are summarized in Table 1.

**Table 1:** Hematological and biochemical laboratory findings at admission. Anti-PF4 antibody testing was not performed due to the unavailability of the assay at our institution, and financial constraints prevented testing at an external laboratory. ALT = alanine aminotransferase; AST = aspartate aminotransferase; GGT = gamma-glutamyl transferase; ALP = alkaline phosphatase; LDH = lactate dehydrogenase; anti-PF4 = anti-platelet factor 4

Parameters	Values	Reference ranges
Platelet count	342,000/μL	150,000–400,000/μL
Hemoglobin	12.5 g/dL	12–16 g/dL
Albumin	44 g/L	35–50 g/L
Glucose	1.88 g/L	0.70–1.10 g/L
Serum creatinine	6.71 mg/L	6–11 mg/L
ALP	59 IU/L	40–130 IU/L
LDH	410 IU/L	120–250 IU/L
GGT	16 IU/L	7–32 IU/L
Urea	0.31 g/L	0.15–0.45 g/L
ALT	25 IU/L	7–35 IU/L
AST	26 IU/L	10–35 IU/L

Laboratory investigations showed a normal platelet count, with no evidence of thrombocytopenia or coagulation abnormality. Liver and renal function tests were also within normal limits. These findings did not support the presence of a thrombotic or coagulation disorder. However, anti-platelet factor 4 (anti-PF4) antibody testing was not performed, representing a limitation of the present case.

The patient was managed in the intensive care unit in a semi-recumbent position and received mechanical ventilation with sedation using propofol and midazolam. Intravenous fluid and electrolyte replacement was provided. Mannitol was administered, and antiepileptic prophylaxis with valproate was initiated. Omeprazole was also administered. Unfortunately, she died two days after hospital admission (18 days after receiving the ChAdOx1 nCoV‑19 vaccine).

In accordance with the national pharmacovigilance system, this case was reported to the National Pharmacovigilance Center through the national post-vaccination adverse event surveillance program.

## Discussion

ICH following SARS-CoV-2 vaccination has been rarely reported in the literature. In a recent review, Panos et al. reported that post-vaccination neurovascular complications include rare hemorrhagic events, most often described in specific contexts and based on limited evidence [4].

The ChAdOx1 nCoV-19 vaccine has been widely used worldwide to control the COVID-19 pandemic. However, rare but serious adverse events have been reported, including neurological complications occurring within days to weeks following vaccination, notably VITT, a condition characterized by thrombosis at unusual sites, thrombocytopenia, and the presence of anti-PF4 antibodies. VITT typically occurs between five and 30 days after ChAdOx1 vaccination and frequently involves cerebral venous sinus thrombosis, which may be complicated by ICH and is associated with a high mortality rate [5,6].

In most reported cases, ICH occurs secondary to venous thrombosis in the setting of VITT. These patients usually present with thrombocytopenia, elevated D-dimer levels, and hypofibrinogenemia, reflecting an underlying prothrombotic state [7].

Regarding our case involving ChAdOx1, it was characterized by several atypical features. The patient developed a fatal ICH 15 days after vaccination, which falls within the expected timeframe for post-vaccination complications. The causality assessment performed using the Naranjo Scale resulted in a score of 4, indicating a possible association between the ChAdOx1 nCoV-19 vaccine and ICH.

Laboratory findings were unremarkable, with a normal platelet count and no evidence of coagulation abnormalities, making VITT unlikely in this context. Such atypical presentations are extremely rare, and only a single case of hemorrhagic stroke following ChAdOx1 vaccination has been reported by de Melo Silva et al., who showed a normal platelet count, normal coagulation parameters, and no vascular abnormalities, suggesting that ICH may occur independently of VITT [3]. A comparison between our case and the similar one reported by de Melo Silva et al. is presented in Table 2.

**Table 2:** Comparison between the present case and a previously reported study. Anti-PF4 = anti-platelet factor 4; VITT = vaccine-induced immune thrombotic thrombocytopenia

Variable	Our case	de Melo Silva et al. (2021) [3]
Age / Sexe	41-year-old woman	57-year-old woman
Vaccine type	ChAdOx1 nCoV-19	ChAdOx1 nCoV-19
Dose number	Not specified	First dose
Time to symptom onset	8–15 days	5 days
Initial symptoms	Headache, eye pain, photophobia, and reduced consciousness	Sweating, paleness, hemiparesis, vomiting, and somnolence
Type of hemorrhage	Intracerebral hemorrhage	Large right frontal intracerebral hematoma with ventricular flooding
Location	Left parietal	Deep frontal lobe
Thrombosis	Not assessed	Absent (no thrombosis on angiography)
Platelet count	Normal	Normal
Coagulation parameters	Normal	Normal
Anti-PF4 antibodies	Not performed	Not reported
VITT likelihood	Unlikely	Unlikely
Outcome	Fatal (death)	Survived with neurological deficit

The underlying mechanisms remain unclear. While VITT is mediated by anti-PF4 antibodies leading to platelet activation and thrombosis, alternative mechanisms may be involved in patients without thrombocytopenia. These may include endothelial dysfunction, inflammation-mediated vascular injury, or other immune-related processes triggered by vaccination, as reported in a few cases of ICH following the BNT162b2 vaccine without evidence of VITT [8-10].

Finally, although the temporal relationship between vaccination and symptom onset may raise the question of a potential association, a causal relationship cannot be established with certainty. ICH is a common neurological emergency with multiple etiologies, including hypertension, vascular malformations, and coagulation disorders, none of which were identified in our case.

## Conclusions

This case describes an atypical ICH occurring within a compatible timeframe following ChAdOx1 nCoV-19 vaccination in the absence of VITT or coagulation abnormalities. It highlights the rarity and heterogeneity of post-vaccination neurological events and reinforces the importance of continued pharmacovigilance through the reporting of well-documented atypical cases. Further studies are needed to better elucidate the underlying mechanisms and clarify potential causal relationships.
